# How Preservice Teachers’ Career Planning Affects Perceived Employability in the Digital Age: A Moderated Mediation Model

**DOI:** 10.3390/bs15091151

**Published:** 2025-08-24

**Authors:** Yangjie Li, Yiwen Fan

**Affiliations:** 1 Jing Hengyi School of Education, Hangzhou Normal University, Hangzhou 311121, China; liyangjie@hznu.edu.cn; 2 Faculty of Education, Beijing Normal University, Beijing 100875, China

**Keywords:** preservice teachers, career planning, job search clarity, digital literacy, perceived employability

## Abstract

With rising employment pressure on preservice teachers in the context of negative population growth and higher education expansion in the world, which have jointly reduced the demand for teachers while increasing the supply of graduates, the digital era offers new opportunities to enhance their employability. This study proposes a moderated mediation model to explore how career planning influences perceived employability, using a sample of 650 respondents. Results show that career planning positively predicts perceived employability, with job search clarity serving as a mediator. Digital literacy significantly moderates this mediation effect. Preservice teachers with higher digital literacy benefit more from career clarity in enhancing perceived employability. These findings suggest that strengthening digital literacy and clarifying career goals are key strategies to improve employability among preservice teachers.

## 1. Introduction

As higher education continues to expand, an increasing number of preservice teachers are joining the workforce, making their self-assessed employability a key focus in recent research (Jenkins & Crawford, 2022; Donald et al., 2024). However, in recent years, the world’s employment environment has faced enormous challenges, and the trend of difficult employment for preservice teachers is not only evident in China, but also increasingly evident in the world (Peng et al., 2024; United Nations Educational, Scientific and Cultural Organization, 2024; Pekmezci & Ertaş, 2024; Xie et al., 2017; Tomlinson, 2012). Due to the negative population growth factor, the number of students enrolled in basic education and the demand for teachers is decreasing, which means that the demand for teachers is also gradually declining, making it more difficult for preservice teachers to find employment (Yu et al., 2024; Mycos Research Institute, 2023; Ministry of Education of the People’s Republic of China, 2023; Rots et al., 2014). With the continuous expansion of higher education, more and more preservice teachers are entering the job market, further increasing the employment pressure they face (Cai, 2013; Barakat & Shields, 2019; Small et al., 2022). At the same time, the advent of the digital age presents a chance to improve the employability of preservice teachers. It is undeniable that enhancing the employability of preservice teachers not only alleviates their employment anxiety but also contributes to improving the quality of their employment. This trend has forced preservice teachers to explore effective ways to enhance their employability. Developing strategies to improve job market competitiveness is essential for preservice educators. Their self-evaluated employment potential serves as a significant indicator of real-world career prospects, since it represents how objective employment capabilities are subjectively interpreted (Vanhercke et al., 2014). Therefore, examining the factors that influence preservice educators’ confidence in their career prospects becomes vital for enhancing both the practical skills and self-assurance they bring to the workforce.

The existing studies have analyzed the factors affecting perceived employability, including goal and behavioral factors (Tavitiyaman et al., 2023; Jung & Li, 2024). These studies have consistently emphasized that job search clarity can be considered as a target factor influencing perceived employability (Yang et al., 2022), which may provide a better explanation of differences in preservice teachers’ perceived employability from a directional level. Furthermore, behavioral factors, such as career planning, can also contribute to an individual’s perceived employability by helping them clarify their employment goals and boosting their employment confidence (Petruzziello et al., 2023; De Cuyper & De Witte, 2011; Forrier et al., 2015; Ma & Chen, 2022). Previous research has identified this factor as a potential determinant of self-perceived career readiness (Wittekind et al., 2010). Nevertheless, few studies have established a link between job search clarity and career planning behaviors and have also investigated the extent to which these behaviors contribute to perceived employability. As two integral components of employment activities, career planning can help job seekers clarify their employment intention goals, thereby further enhancing their employment confidence (Shury et al., 2017; Clements & Kamau, 2018). Career planning proves especially crucial for preservice educators experiencing uncertainty regarding their professional aspirations and self-assurance in the job market. The current research highlights the importance of incorporating career decision-making clarity when examining how vocational preparation influences self-assessed career readiness.

Moreover, the concept of goal-setting theory posits that the formulation of goals can influence the outcome of an endeavor by prompting the evocation, discovery, or utilization of task-related knowledge or strategies (Wood, 1990). Based on goal-setting theory, we hypothesize that preservice teachers’ job search clarity serves as a goal factor that mediates the relationship between career planning and perceived employability. Furthermore, digital literacy is hypothesized to moderate the strength of this mediating pathway, such that the indirect effect is stronger for those with higher digital literacy. Specifically, it can be divided into these research questions: (1) To what extent does career planning directly predict preservice teachers’ perceived employability in the digital age? (2) Does job search clarity function as a mediating mechanism through which career planning enhances perceived employability? (3) Does digital literacy moderate this mediation pathway, such that the indirect effect of career planning on perceived employability via job search clarity becomes stronger when digital literacy is higher? The ability to utilize digital technologies is an increasingly significant indicator of an individual’s perceived employability (Bejaković & Mrnjavac, 2020). It has been demonstrated that in the digital age, university students with higher digital literacy are better able to develop employment strategies around goals, capture and analyze relevant information (Gao, 2023), and thus enhance their employment confidence, especially among the group of job seekers with clear goals (Leung, 2022). This indicates that the digital literacy of university students plays a moderating role in perceived employability. However, these studies have not yet fully elucidated the moderating effect of preservice teachers’ digital literacy on perceived employability. Moreover, it remains uncertain how the interplay between preservice teachers’ job search clarity and digital literacy influences their perceived employability.

Building on these theoretical foundations, the present research develops a moderated mediation model to examine how preservice educators’ career preparation affects their self-evaluated professional competencies within digital environments. This investigation aims to elucidate the multidimensional pathways—encompassing vocational preparation strategies, career decision-making precision, and technological competencies—that contribute to strengthening preservice teachers’ professional self-efficacy. We focus on preservice teachers because, in addition to the expansion of higher education, the shrinking school-age population has substantially reduced the demand for teachers, intensifying the employment challenges they face. From the theoretical perspective of the IEM model, this study focuses on the perceived employability of preservice teachers and explores the relationship between career planning, job-seeking clarity, and digital literacy. It is hoped that teaching and technical strategies can be designed for pre-service teachers with different levels of digital literacy to enhance their perceived employability, take advantage of the digital dividend, and expand their employment methods and ideas. 

## 2. Theoretical Framework and Assumptions

### 2.1. Perceived Employability and the IEM Model

Perceived employability, defined as individuals’ subjective evaluation of their professional marketability (Vanhercke et al., 2014), constitutes a pivotal determinant affecting graduates’ transition into the workforce. This self-perception is shaped by multiple determinants, including occupation-specific competencies, vocational preparation strategies, and related factors, with these predictors manifesting through job-related knowledge and skills, career planning behaviors, etc. (Rothwell & Arnold, 2007; Ayala Calvo & Manzano García, 2021), and these influences are reflected in the Integrated Model of Graduate Employability (IEM). The IEM model, initially proposed by Clarke (2018), systematically presents the effects of human capital, social capital, individual behaviors, individual attributes, and the labor market on graduates’ perceived employability. Among these factors, human capital encompasses skills, abilities, and work experience. In the digital age, digital literacy is a key factor in human capital, which can help preservice teachers find jobs more efficiently and accurately. Moreover, individual behaviors are typically regarded as career-related behaviors (Fugate et al., 2004; Gould, 1979), of which the more typical one is career planning. Earlier systematic career planning has been demonstrated to have a significant impact on job search clarity (Zikic & Saks, 2009), which can further enhance perceived employability (Yang et al., 2022). Accordingly, Clarke’s holistic framework of graduate employability serves as the theoretical foundation for this research. Building on this, prior studies have suggested that career planning enhances job search clarity, which, in turn, strengthens perceived employability, while digital literacy functions as a crucial resource that interacts with both career planning and job clarity to shape employability outcomes (Caroline et al., 2025). Using these, this study facilitates an investigation into how career planning influences preservice teachers’ perceived employability, as well as the contributory role of digital literacy within this relationship. On this basis, this study put forward the following hypotheses:

**H1.**  *Perceived employability (a) is positively related to career planning (b), job search clarity (c), and digital literacy (d)*.

### 2.2. Career Planning and Perceived Employability

Career planning is considered to be a pre-arranged, systematic plan for future career development by an individual (Gould, 1979). The IEM model posits that proactive career development strategies demonstrate greater predictive power for self-evaluated professional readiness than institutional or socioeconomic factors (Okay-Somerville & Scholarios, 2017). Longitudinal studies demonstrate that structured career preparation interventions significantly enhance graduates’ self-assessed employment competence throughout their school-to-work transition period (Chughtai, 2019; Jackson & Tomlinson, 2020). Praskova et al. (2015) also suggests that both career exploration and career planning are associated with higher perceived employability. In essence, career planning enables graduates to be better prepared for their careers, to set job search goals at an earlier stage, and to have higher subjective perceptions of employability. However, most previous studies have focused on the broader group of university students, with less research on the smaller group of preservice teachers. Based on these findings, this study proposes the following hypothesis:

**H2.**  *Career planning will positively predict perceived employability*.

### 2.3. The Mediating Effect of Job Search Clarity

Locke and Latham’s (1990) goal-setting theory demonstrates that establishing specific, challenging objectives enhances performance, a principle applicable to career development, where structured goal-setting strengthens professional self-efficacy (Clements & Kamau, 2018). Career decision-making clarity—defined as the precision of one’s occupational targets (Wanberg et al., 2002)—correlates with proactive career preparation, supported by evidence that multidimensional career information access refines goal specificity (Benati et al., 2023). Notably, occupational clarity moderates the human capital–employability link (Yang et al., 2022), though research gaps persist regarding its mediating role in competency development during job transitions. However, existing studies have less research on the mediating mechanism of job search clarity, and the hypothesis that career planning affects perceived employability through the mediating effect of job search clarity remains to be tested. Based on these findings, this study proposes the following hypothesis:

**H3.**  *Job search clarity mediates between career planning and perceived employability*.

### 2.4. The Moderating Effect of Digital Literacy

In the IEM model, both individual behavior and human capital are vital factors influencing perceived employability. Individual behavior refers to career planning, and human capital refers to digital literacy, focusing on how job search clarity affects perceived employability through digital literacy. In the digital age, digital literacy plays an increasingly important role, and it encompasses the ability to effectively utilize digital tools for information processing, critical evaluation, content creation, and communication (Martin, 2006; Kereluik et al., 2013; Anthonysamy et al., 2020). In general, job search clarity has an impact on perceived employability. However, in the digital age, particularly in digitized workplaces, those with advanced technical competencies exhibit stronger career adaptability (Karaoglu et al., 2022; Green, 2017; Bejaković & Mrnjavac, 2020). Moreover, while individuals who engage in career planning may enhance their job search clarity, it is not a guaranteed pathway to perceived employability, particularly in the context of rapid labor market shifts (Benati et al., 2023; Männasoo et al., 2023). It is therefore important to introduce digital literacy. It is proposed that there is an interaction between preservice teachers’ job search clarity and digital literacy on perceived employability. Preservice teachers’ perceived employability will be highest when both job search clarity and digital literacy are high and lowest when both factors are low. This is because a lack of digital literacy makes it challenging for an individual to search for relevant career information and to develop an awareness of their own employability, particularly if they lack clarity about their career aspirations. Therefore, this study proposes the following hypothesis:

**H4.**  *Digital literacy moderates the relationship between job search clarity and perceived employability*.

**Figure 1:**
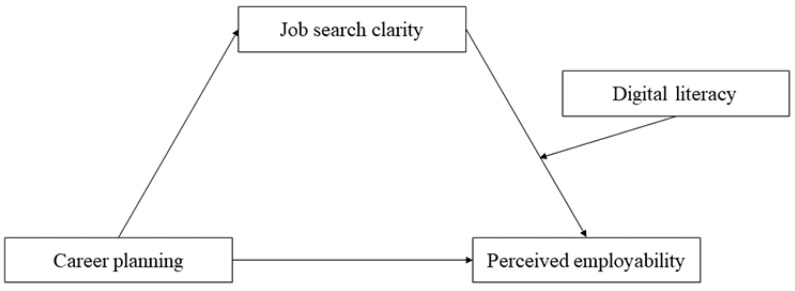
The proposed research model on career planning, perceived employability, job search clarity, and digital literacy.

Literature reviews suggest that preservice teachers’ career planning is not only directly related to their perceived employability but also indirectly related to their perceived employability through the mediating factor of job search clarity and the moderating factor of digital literacy. We aimed to explore the relationships and functional mechanisms between preservice teachers’ career planning, perceived employability, job search clarity, and digital literacy. The relationships between these constructs have been integrated into a conceptual model, depicted in Figure 1.

## 3. Methods

### 3.1. Participants

The participants in this study were from Hangzhou, which is relatively highly digitized and provides an optimal digital environment for the employment of preservice teachers. All participants were preservice teachers from Hangzhou Normal University, where the employment rate of preservice teachers is close to the national average. Meanwhile, due to negative population growth and the expansion of higher education, preservice teachers at the university are facing employment difficulties, which is consistent with the current employment situation in China and even the world (Peng et al., 2024; Xie et al., 2017). In total, 650 valid responses were obtained. All participants were volunteers and signed the informed consent form. The proportion of valid responses from each grade of college was as follows: 26.3% from grade 1, 23.2% from grade 2, 32.2% from grade 3, and 18.3% from grade 4.

### 3.2. Instrumentation and Validation

The questionnaire used in this research consisted of two major components. The first section was demographic information, including gender, grade level, and the type of institution. The second section was career planning, job search clarity, digital literacy, and perceived employability. All measurement tools were drawn from well-established scales that have demonstrated strong reliability and validity in prior scholarly work. All items were measured on a five-point Likert scale, ranging from “completely disagree” to “completely agree”. Data collection was conducted in December 2023, followed by data analysis from January to February 2024. Both the questionnaire content and research procedures received formal approval from the Ethics Committee of Hangzhou Normal University via Hengyi College of Education. The principles of anonymity, independence, and confidentiality of the survey were strictly adhered to during the data collection process, with the data collected via a questionnaire. The questionnaire was distributed online via the Wenjuanxing platform. A stratified sampling approach was adopted, with surveys sent to students from each undergraduate grade level (freshman to senior). A total of 650 valid responses were collected, ensuring broad representation across preservice teachers’ academic stages.

In terms of career planning, we adopted five items from Jackson and Tomlinson (2020). Job search clarity was measured using five items developed by Côté et al. (2006). A sample item was: ‘I have set a goal for the type of job I want to have when I graduate’. Participants’ perceived employability was assessed through their post-graduation occupational goals. Professional self-perception was evaluated using Berntson and Marklund’s (2007) five-item instrument. Digital literacy was measured via W. Ng’s (2012) 10-item scale, which evaluates educational technology proficiency across technical, cognitive, and social–emotional domains. A sample item was: ‘I have good ICT skills’. Meanwhile, the reliability test was conducted, and the Cronbach’s alpha values applied to career planning, job search clarity, digital literacy, and perceived employability were 0.91, 0.92, 0.96, and 0.92, demonstrating a high level of internal consistency and confirming the strong reliability of the instrument. Confirmatory factor analysis was performed using AMOS26.0. The results of the statistical analysis tool were as follows: x^2^/df = 3.922 < 5, NFI = 0.928 > 0.9, IFI = 0.946 > 0.9, TLI = 0.939 > 0.9, and CFI = 0.945 > 0.9. All the fitting indicators were better than the recommended values, indicating that the questionnaire structure was clear and had good structural validity.

### 3.3. Data Analysis Method

Statistical analyses were performed using two computational tools: IBM SPSS (Version 27) for preliminary examinations and Hayes’ PROCESS (Version 4.0) for advanced modeling. The investigation progressed through three sequential stages: (1) verification of measurement reliability and validity, (2) computation of fundamental descriptive statistics and intervariable correlations, and (3) testing of conditional process effects through moderated mediation analysis. 

## 4. Results

### 4.1. Descriptive Statistics

Data screening revealed that there were no outliers in our data. According to Table 1, the Pearson correlation study showed that career planning, job search clarity, digital literacy, and preservice teachers’ perceived employability were significantly and positively correlated with each other. Therefore, H1 is supported by the results of the correlation analysis of this study.

### 4.2. The Mediating Role of Job Search Clarity

This study used Model 4 in PROCESS macro version 4 to examine the mediating role of digital literacy on the relationship between job search clarity and the perceived employability of preservice teachers. The results are shown in Table 2 and Table 3. Career planning was a significant direct positive predictor of preservice teachers’ perceived employability (β = 0.60, t = 15.73, and *p* < 0.001). The direct positive effect remained significant with the addition of the mediating variable (β = 0.47, t = −11.42, and *p* < 0.001). Furthermore, the 95% bootstrap confidence interval for the mediating role of job search clarity excluded zero, confirming its statistical significance. The direct pathway from career planning to perceived employability contributed 79.66% to the total effect, while the indirect pathway through job search clarity accounted for 20.33%. Thus, the findings provide empirical support for Hypotheses 2 and 3.

All variables included in the model were standardized, and the same approach was consistently applied throughout the analysis. The 95% confidence intervals were estimated using 5000 bootstrap resamples, which were uniformly implemented across all subsequent analyses.

### 4.3. Moderated Mediated Model Testing

Hayes’ PROCESS analysis (Model 59, v4.0) revealed three key findings, as illustrated in Table 4, First, digital competence significantly predicted employment self-efficacy (β = 0.51, t = 10.63, *p* < 0.001). Second, career decision clarity positively influenced perceived employability (β = 0.16, t = 4.66, *p* < 0.001). Most importantly, their interaction term was statistically significant (β = 0.10, t = 2.76, *p* < 0.01), indicating that technological proficiency enhances the positive impact of vocational clarity on professional confidence, thus validating our fourth hypothesis.

**Figure 2:**
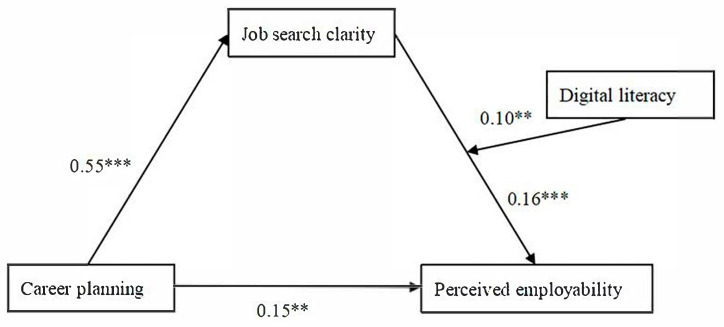
Results of the model testing on career planning, perceived employability, job search clarity, and digital literacy. ** *p* < 0.01, and *** *p* < 0.001.

As illustrated in Table 5, the mediating role of job search clarity varied depending on the level of digital literacy. When participants demonstrated moderate digital literacy, the mediation effect was significant (effect = 0.08, with a 95% bootstrap confidence interval excluding zero), and this effect was even stronger at high levels of digital literacy (effect = 0.12, 95% CI not including zero). In contrast, for those with low digital literacy, the mediation effect was not statistically significant (effect = 0.05, 95% CI including zero). These findings indicate that the mediating influence of job search clarity between career planning and perceived employability becomes more pronounced as digital literacy increases. Overall, the latter segment of the pathway—namely, ‘career planning → job search clarity → perceived employability’—is significantly moderated by digital literacy, supporting the formulation of a moderated mediation framework. The hypothesized model integrating mediation and moderation effects received statistical support. Figure 2 illustrates the path coefficients and interaction patterns derived from the analysis. 

**Figure 3:**
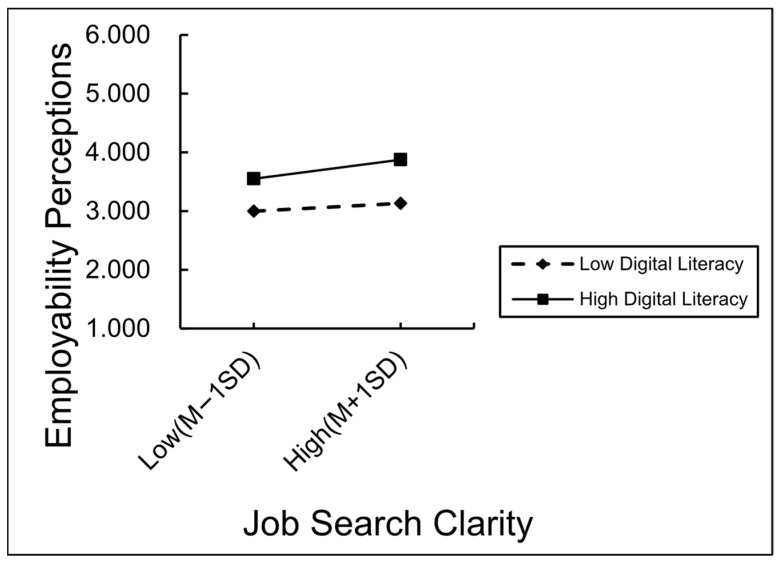
The moderating mechanism of digital literacy.

To further reveal the regulatory mechanism of digital literacy, we conducted a subgroup analysis, defining individuals with a digital literacy score one standard deviation above the average as the “high digital literacy group” and those with a score one standard deviation below the average as the “low digital literacy group”. The predictive effect of job search clarity on perceived employability was then analyzed separately for each subgroup. As presented in Figure 3, job search clarity significantly predicted perceived employability in both cases. Notably, the predictive strength was greater in the low digital literacy group (β = 0.09, t = 2.07, *p* < 0.05), compared to the high digital literacy group (β = 0.22, t = 5.88, *p* < 0.001). These findings indicate that the influence of job search clarity on perceived employability tends to be more pronounced among individuals with lower digital literacy, suggesting that digital proficiency may buffer or diversify the pathways through which employability is enhanced.

## 5. Discussion and Implications

To enhance the employability of preservice teachers, this study proposed a moderated mediation model with the perceived employability of preservice teachers at different grades. Through linear regression modeling, we assessed the relationships among preservice teachers’ career planning, job search clarity, digital literacy, and perceived employability, providing the latest research findings in this field, which have important practical significance for higher education. Meaningful findings are discussed and presented below.

First, career planning enhances employability through job search clarity, and path analysis identified career decision clarity as a full mediator in the relationship between vocational preparation and employment self-efficacy. However, the empirical survey of this study shows that preservice teachers believe that their career planning and job search clarity have not yet reached a high level, which suggests that there is still much room for improvement in preservice teachers’ preparation for employment and clarity of goals in the pre-service stage, a finding that is consistent with that of college students in other majors (Yanik et al., 2012; Zhu et al., 2021). This requires preservice teachers to fully mobilize themselves to explore their career goals and development paths as early as possible and to continuously adjust their goals and plans during their teacher education. Effective strategies include constructing preservice teachers’ career planning communities, building preservice teachers’ employment support systems, and the creation of feedback mechanisms for their career planning (Renn et al., 2014; Reese & Miller, 2006; Zhao & Wu, 2022). These are designed to address the lack of awareness among preservice teachers of the importance of individual career planning and the ambiguity of their job-seeking goals.

Second, digital literacy strengthens the impact of job search clarity on employability. This indicates that the impact of job search clarity is relatively limited when preservice teachers exhibit low levels of digital literacy. Among digitally literate preservice teachers, job search clarity is essential for employability, aligning with goal-setting theory, which posits that goal attainment relies on mobilizing task-relevant strategies and knowledge (Wood, 1990). When preservice teachers identify clearer job search goals, they can access, analyze, integrate, and creatively use employment information with the help of digital technology (Ha & Kim, 2024), which has a greater impact on their perceived employability. More specifically, preservice teachers with higher levels of digital literacy perceive an increase in employability based on clearer job search goals. This deepens the perception that digital literacy of preservice teachers affects job competitiveness and job quality, based on the validation of existing research. Furthermore, the implications of our study go beyond the Chinese teacher education context. While digital literacy is a prominent factor among so-called “digital natives”, our findings suggest that even within this cohort, digital proficiency varies significantly, influencing employability trajectories. This raises critical questions for global teacher education systems in contexts where digital exposure is limited or uneven. For instance, in many low-income or rural teacher education programs globally, access to digital infrastructure is restricted. Thus, targeted training in digital competencies should not be assumed but intentionally designed into teacher preparation curricula—especially in systems transitioning into hybrid or tech-integrated pedagogies.

In addition to the above conclusions, this study has also made contributions in the theoretical aspect. Our study not only validates the IEM model but, in a broader sense, extends it from the general context of digital literacy research to a more context-specific one. The IEM model has systematically presented the impact of human capital on graduates’ perceived employability, and preservice teachers can improve their perceived employability by improving their digital literacy by becoming clearer about their employment goals and by planning and preparing accordingly. Empirical evidence positions human capital as fundamental to employment confidence development (P. M. Ng et al., 2022). Building on Becker’s (1993) theoretical framework, which defines human capital as knowledge-based productive assets influencing vocational advancement, the digital transformation era has elevated technological literacy to a central position in modern human capital formation (Van Laar et al., 2017; Anthonysamy et al., 2020). Preservice teachers who are highly digitally literate tend to have an advantage in the labor market, not only by having easy access to more employment information and participating more fully in employment-related online activities but also by being able to self-educate in digital environments that provide new tools for optimizing formal education, and their perceived self-employability is more related to cognitive changes gained with the help of digital technology (Gao, 2023; Ramos-Monge et al., 2023). This study aims to enhance the digital literacy of preservice teachers, reduce the problem of information asymmetry, and alleviate the high employment pressure on preservice teachers. Therefore, this study places great emphasis on digital literacy as a moderating variable. Preservice teachers do not merely obtain employment opportunities through the job information released by the school or through job fairs. Instead, they acquire employment information from various digital channels, increase their job opportunities, and enhance their confidence in employment.

Our study adds to the existing research by finding that digital literacy plays a moderating role in understanding preservice teachers’ perceived employability. Our findings remind us that with the ever-changing external environment and the advent of the digital age, new demands are being placed on the employability of preservice teachers, and new opportunities are being provided for the development of preservice teachers’ employability (Şensoy Murt & Erdur-Baker, 2023; Martínez-Moreno & Petko, 2023). In other words, when preservice teachers have formulated their initial job search goals through career planning, they can enhance their perceived employability through digital literacy and diversify their approaches. First, in response to the Chinese government’s goal of building a high-quality, professional, and innovative teaching force, preservice teachers are expected to stay abreast of evolving teacher training requirements, continuously refine their job search objectives, and strategically align their efforts by leveraging digital technologies. Second, digital tools can significantly expand preservice teachers’ employment horizons and facilitate simulated job search experiences, enabling them to move beyond the limitations of their own experience and imagination. Third, it is essential to enhance preservice teachers’ digital literacy through a comprehensive and systematic cultivation framework, which, in turn, contributes meaningfully to improving their perceived employability. Fourth, based on the findings, teacher training institutions should incorporate structured digital literacy modules and career counseling services into their curricula. For example, digital job search simulations can help students practice goal-setting and information analysis. Tailored digital interventions can also support students with lower digital proficiency to strengthen their employability pathways. Fifth, this study contributes to existing literature by critically reflecting on how digital literacy should be more than a general recommendation; rather, it needs to be operationalized within teacher education curricula. For example, institutions should move beyond offering basic ICT training and instead integrate goal-oriented digital employability programs. These might include AI-enhanced career tools, adaptive digital job search simulations, or scaffolded digital portfolios that align with students’ evolving career goals. Moreover, our findings suggest that digital literacy interventions should be differentiated based on students’ baseline proficiency, with targeted support for those at lower levels to prevent digital exclusion. These implications are particularly relevant for low-resource or unequal digital access contexts, where bridging the digital gap is essential for equitable employability development.

## 6. Conclusions, Limitation, and Future Research

In summary, this study developed a novel moderated mediation framework to investigate how career planning influences perceived employability through job search clarity, with digital literacy serving as a contextual enhancer. Results indicate that systematic career development enhances occupational goal precision, which subsequently strengthens employment confidence. Notably, the facilitating effect of job search clarity on perceived employability was perceived to be negatively moderated by preservice teachers’ digital literacy. There is a need for preservice teachers to increase the frequency of their practice in digital space, to grasp employment information, and to prepare for employment through a variety of digital means based on clear job search goals and thus enhance perceived employability. The findings of this research offer valuable insights for higher education institutions aiming to enhance employment education for preservice teachers in response to the ongoing digital transformation in the sector. By identifying the moderating effect of digital literacy, this study also advances the theoretical development of the Individual Employability Model (IEM). Specifically, it empirically examines how two core dimensions of the model—employment behavior management and competence-based literacy—affect perceived employability (Clarke, 2018), which are operationalized in this study as “career planning” and “digital literacy”, respectively.

This study primarily investigates how career planning influences preservice teachers’ perceived employability. However, the reliance on self-report instruments introduces potential response biases, particularly regarding perceived employability. Individuals may overestimate or underestimate their employment readiness due to social desirability, self-enhancement, or self-doubt, which may not align with objective labor market outcomes. For instance, students with stronger self-efficacy might report higher job search clarity and career planning, not necessarily because of actual planning behaviors but due to a more optimistic self-perception. Therefore, future research should consider incorporating additional variables and employing longitudinal designs to better capture preservice teachers’ real-world employment-related behaviors in digital contexts. Moreover, the participants in this study were drawn from various grade levels, reflecting diverse levels of digital literacy, online job preparedness, and psychological attributes. One key limitation of this study is that the sample was drawn exclusively from Hangzhou Normal University. Although the university is representative in terms of digital conditions and teacher training, the findings may not generalize to other regions or countries. Future research should conduct cross-cultural comparative studies to explore how cultural and regional differences influence the role of digital literacy in perceived employability. The conclusion drawn from the data analysis of this study is that preservice teachers with strong career planning abilities influence their perceived employability. However, there may also be a reverse causal relationship, that is, preservice teachers with strong perceived employability may feed back into their career planning behavior. We will continue to conduct research in the future, and future studies are encouraged to adopt experimental or longitudinal designs to validate the causal impact of digital literacy interventions on perceived employability.

## Figures and Tables

**Table 1:** Mean, standard deviation, and correlation coefficient of each variable.

	M	S.D.	CP	JSC	PE	DL
CP	3.77	0.63	-			
JSC	3.69	0.74	0.463 **	-		
PE	3.41	0.71	0.526 **	0.426 **	-	
DL	3.63	0.64	0.708 **	0.424 **	0.615 **	-

** *p* < 0.01, double tail; under-triangle is the correlation of the latent variables; CP = career planning, JSC = job search clarity, PE = perceived employability, DL = digital literacy.

**Table 2:** Mediating effect of job search clarity.

Variable	Equation 1: Perceived Employability			Equation 2: Perceived Employability		
	β	Boot SE	T	β	Boot SE	t
Constant	1.16	0.15	7.98 ***	0.79	0.15	5.2 ***
Career Planning	0.60	0.04	15.73 ***	0.47	0.04	11.42 ***
Job Search Clarity				0.22	0.04	6.35 ***
R	0.28			0.32		
F	247.59			151.47		

*** *p* < 0.001.

**Table 3:** Total effects, direct effects, and mediating effects.

	Effect	Boot SE	Bootstrap 95% CI		Relative Effect Size (%)
			Lower	Upper	
The total effect	0.59	0.04	0.52	0.67	
Direct effect	0.47	0.04	0.39	0.56	79.66
Mediating effect of Job Search Clarity	0.12	0.03	0.07	0.18	20.33

**Table 4:** Test results of moderated mediating effects.

Variable	Equation 1: Job Search Clarity			Equation 2: Perceived Employability		
	β	Boot SE	t	β	Boot SE	t
Constant	−2.06	0.16	−13.12 ***	2.82	0.19	14.89 ***
Career Planning	0.55	0.04	13.3 ***	0.15	0.05	3.02 **
Digital literacy				0.51	0.05	10.63 ***
Job Search Clarity				0.16	0.03	4.66 ***
Job Search Clarity × Digital literacy				0.10	0.04	2.76 **
R	0.22			0.43		
F	176.94			119.27		

** *p* < 0.01, and *** *p* < 0.001.

**Table 5:** The mediating effect of job search clarity under different levels of digital literacy.

	Perceived Interest	Effect	Boot SE	Bootstrap 95% CI	
				Lower	Upper
Mediating effects of job search clarity	eff1(M − 1SD)	0.05	0.02	−0.02	0.11
	eff2(M)	0.08	0.03	0.03	0.14
	eff3(M + 1SD)	0.12	0.04	0.05	0.19
Comparison of the mediating effects of job search clarity	eff2–eff1	0.04	0.02	−0.00	0.09
	eff3–eff1	0.07	0.04	−0.00	0.16
	eff3–eff2	0.04	0.02	−0.00	0.08

## Data Availability

The raw data supporting the conclusions of this article will be made available by the authors upon request.
